# Efficacy of avapritinib versus best available therapy in the treatment of advanced systemic mastocytosis

**DOI:** 10.1038/s41375-022-01615-z

**Published:** 2022-07-05

**Authors:** Andreas Reiter, Jason Gotlib, Iván Álvarez-Twose, Deepti H. Radia, Johannes Lübke, Priyanka J. Bobbili, Aolin Wang, Chelsea Norregaard, Saša Dimitrijevic, Erin Sullivan, Melinda Louie-Gao, Juliana Schwaab, Ilene A. Galinsky, Cecelia Perkins, Wolfgang R. Sperr, Priya Sriskandarajah, Andi Chin, Selvam R. Sendhil, Mei Sheng Duh, Peter Valent, Daniel J. DeAngelo

**Affiliations:** 1 Department of Hematology and Oncology, University Hospital Mannheim, Mannheim, Germany; 2 Stanford Cancer Institute/Stanford University School of Medicine, Stanford, CA USA; 3 Institute of Mastocytosis Studies of Castilla La Mancha (CLMast)—Spanish Reference Center (CSUR) for Mastocytosis and CIBERONC, Virgen del Valle Hospital, Toledo, Spain; 4 Guy’s and St Thomas’ NHS Foundation Trust of Guy’s Hospital, London, UK; 5 Analysis Group, Inc., Boston, MA USA; 6 Blueprint Medicines Corporation, Cambridge, MA USA; 7 Blueprint Medicines Corporation, Zug, Switzerland; 8 Department of Medical Oncology, Dana-Farber Cancer Institute, Boston, MA USA; 9 Department of Internal Medicine I, Division of Hematology and Hemostaseology, Medical University of Vienna, Vienna, Austria; 10 Ludwig Boltzmann Institute for Hematology and Oncology, Medical University of Vienna, Vienna, Austria

**Keywords:** Cancer therapy, Targeted therapies

## Abstract

Advanced systemic mastocytosis (AdvSM) is a rare myeloid neoplasm associated with poor overall survival (OS). This study (NCT04695431) compared clinical outcomes between patients with AdvSM treated with avapritinib in the Phase 1 EXPLORER (NCT0256198) and Phase 2 PATHFINDER (NCT03580655) trials (*N* = 176) and patients treated with best available therapy (BAT; *N* = 141). A multi-center, observational, retrospective chart review study was conducted at six study sites (four European, two American) to collect data from patients with AdvSM who received BAT; these data were pooled with data from EXPLORER and PATHFINDER. Comparisons between outcomes of OS, duration of treatment (DOT), and maximum reduction in serum tryptase were conducted between the treatment cohorts, with adjustment for key covariates. The results indicated that the avapritinib cohort had significantly better survival (adjusted hazard ratio (HR) (95% confidence interval (CI)): 0.48 (0.29, 0.79); *p* = 0.004) and significantly longer DOT (HR: 0.36 (0.26, 0.51); *p* < 0.001) compared to the BAT cohort. Additionally, the mean difference in percentage maximum reduction in serum tryptase levels was 60.3% greater in the avapritinib cohort (95% CI: −72.8, −47.9; *p* < 0.001). With no randomized controlled trials comparing avapritinib to BAT, these data offer crucial insights into the improved efficacy of avapritinib for the treatment of AdvSM.

## Introduction

Advanced systemic mastocytosis (AdvSM) is a rare myeloid neoplasm characterized by accumulation of neoplastic mast cells in various tissues and organs [1–4], often leading to progressive organ damage, mainly manifesting as cytopenias of one or more hematopoietic lineage(s) and dysfunction of gastrointestinal organs [5]. The World Health Organization defines three subtypes of AdvSM: aggressive systemic mastocytosis (ASM), SM with an associated hematologic neoplasm (SM-AHN), and mast cell leukemia (MCL) [6]. Patients with AdvSM have a poor prognosis, with a median overall survival (OS) of ~3.5 years for ASM, 2 years for SM-AHN, and 0.5–2 years for MCL [7–10].

As the majority (>90%) of patients with AdvSM carry a *KIT* D816V mutation [11], recent therapeutic advances have focused on KIT inhibitors [12]. Treatment options for patients with AdvSM include the multikinase KIT inhibitor midostaurin, for which efficacy and safety has been reported in several clinical trials and observational studies [9, 13–18]. In addition, imatinib is a treatment option for the limited indication of ASM patients who are *KIT* D816V-negative or with unknown *KIT* mutation status [19]. Commonly used off-label cytoreductive therapies include cladribine [13, 20–23] and interferon alfa [22, 24]. For treatment-resistant patients and those with rapidly progressive disease after tyrosine kinase inhibitor (TKI) treatment, multiagent chemotherapy and subsequent allogenic hematopoietic stem cell transplantation (HSCT) are considerations [25]. Indeed, HSCT is the only established curative treatment option for these patients.

Avapritinib, a highly selective and potent inhibitor of D816V-mutated KIT, was evaluated in adults with centrally confirmed AdvSM in two multi-center, single-arm, open-label clinical trials, the Phase I EXPLORER trial (ClinicalTrials.gov Identifier: NCT02561988) [26] and Phase II PATHFINDER trial (NCT03580655) [27]. Analysis of data from 69 patients with AdvSM in EXPLORER reported an estimated 24-month OS rate of 76% (95% confidence interval (CI), 64–87%) and that 99% of patients achieved ≥50% reduction from baseline in serum tryptase (a common marker of mast cell activation) [26]. Similarly, in a pre-specified interim analysis of 62 patients who received avapritinib primarily at a starting dose of 200 mg daily from PATHFINDER, the estimated 12-month OS rate was 86% and 93% of patients achieved ≥50% reduction from baseline in serum tryptase [27].

Given the single-arm design of EXPLORER and PATHFINDER, a direct comparison of avapritinib to alternative therapies for AdvSM is not feasible in the context of a controlled clinical trial. However, comparing the efficacy of avapritinib with that of existing therapies for AdvSM is essential to inform clinical decision making. To address this need, the current study compared the efficacy of avapritinib to a real-world cohort of similar patients receiving best available therapy (BAT) for AdvSM.

## Methods

### Study population

#### Clinical trial data (avapritinib cohort)

Individual patient data as of the April 20, 2021, data cut-off from the Phase I EXPLORER and Phase II PATHFINDER trials (data on file, Blueprint Medicines Corporation) were used in this analysis. In EXPLORER, the starting dose of avapritinib was escalated from 30 to 400 mg daily while in PATHFINDER, all but two patients received 200 mg daily.

#### Real-world data (BAT cohort)

A multi-center, observational, retrospective chart review study was conducted to generate real-world data on BAT for AdvSM. Longitudinal, individual-level data were collected via medical chart abstraction on eligible patients with AdvSM who received systemic treatment at the following Centers of Excellence for the treatment of AdvSM: Dana-Farber Cancer Institute (United States [US]), Guy’s and St Thomas’ NHS Foundation Trust (United Kingdom), Hospital Virgen del Valle (Spain), Medical University of Vienna (Austria), University Hospital Mannheim (Germany), and the Stanford Cancer Institute (US). De-identified data from eligible patients at these sites were abstracted from medical records into a standardized, structured, electronic case report form from March 26, 2021, to October 4, 2021. Site research personnel were provided training on the study protocol and case report form, and data collection was followed by a query resolution process. Ethics Committee approvals were gained at each study site.

### Sample selection

Patients receiving treatment with BAT for AdvSM were identified based on inclusion and exclusion criteria similar to those from EXPLORER and PATHFINDER (full list of criteria provided in Supplementary Table 1). Adults (aged ≥ 18 years) with a diagnosis of AdvSM and documented subtype in their chart (ASM, SM-AHN, or MCL), and who had received ≥1 line of systemic therapy (not necessarily as first line (1L)) for AdvSM at a participating site on or after January 1, 2009, were included. If a patient received multiple lines of therapy at a participating site, data on all available therapies were collected and analyzed (i.e., the patient could contribute more than one line of therapy to the analysis). The date of initiation of each line of therapy at the participating site was defined as the index date.

Patients in the BAT cohort were excluded if they had a history of another primary malignancy that was diagnosed or required therapy within 3 years before the index date, except for completely resected basal cell and squamous cell skin cancer, curatively treated localized prostate cancer, and completely resected carcinoma in situ at any site, or if they received avapritinib as the first therapy for AdvSM.

### Study endpoints

The primary endpoint was OS, defined for the BAT cohort as the time interval between initiation of each line of therapy and death due to any cause, and for the avapritinib cohort as the time interval between the first dose of avapritinib and death due to any cause. If alive at study end, patients were censored at the date of last contact (BAT cohort), or at the last known date alive (avapritinib cohort). Secondary endpoints included (1) duration of therapy (DOT), defined as the time interval between initiation of each therapy to discontinuation for any reason; (2) change in serum tryptase levels from baseline to 2 months (to correspond to the day 1 of cycle 3 assessment of serum tryptase in EXPLORER and PATHFINDER and to maximize the sample size), and (3) maximum reduction in serum tryptase levels from baseline. Other response endpoints such as complete and partial response and clinical improvement were not included due to lack of uniform assessment criteria in non-protocol clinical practice. Adverse events (AEs) that resulted in treatment modification or discontinuation, hospitalization, or death according to the responsible physician’s evaluation were reported for the BAT cohort only, as comparable definitions of AEs were not available in EXPLORER and PATHFINDER.

### Baseline covariates

Multiple prognostic factors for survival and clinical outcomes in patients with AdvSM, as well as confounders for the effect of treatment on outcomes, were considered [12–14, 16, 28, 29]. These a priori-defined key adjustment covariates, informed through clinical input as well as prognostic scores such as the mutation-adjusted risk score [14] and the International Prognostic Scoring System in mastocytosis [30], included age; sex; region (North America or Europe); European Cooperative Oncology Group performance status score; AdvSM subtype (SM-AHN, ASM, or MCL, assessed at the last diagnosis evaluation prior to or on the initiation of an included line of therapy); presence of skin involvement (including reported mastocytosis in the skin or urticaria pigmentosa); number and types of prior lines received (TKI, cytoreductive, or biologic or other systemic therapy); presence of anemia (hemoglobin <10 g/dl), thrombocytopenia (platelet count <100 × 10^9^/l), or leukocyte count ≥16 × 10^9^/l; serum tryptase level ≥125 ng/ml; and presence and number of mutations within the *SRSF2*/*ASXL1*/*RUNX1* gene panel [14, 30].

### Statistical analyses

AEs were evaluated in all BAT patients meeting the above inclusion and exclusion criteria. In comparative analyses of the primary and secondary efficacy endpoints, BAT patients were excluded if they had missing data on a key adjustment covariate. Patients in the safety populations of EXPLORER and PATHFINDER (i.e., received at least one dose of avapritinib and had confirmed AdvSM subtype based on adjudication by the trial Response Assessment Committee (RAC)) were included in the comparative analyses. RAC-response evaluable (RAC-RE) patients from PATHFINDER were included in one subgroup analysis. An integrated dataset containing patient-level data from the avapritinib and BAT cohorts was created, with harmonization between the definitions of outcomes and key covariates.

#### Cohort characteristics and covariates

Descriptive analyses were used to summarize therapies received by patients in the BAT cohort, as well as key covariates in both cohorts. Means, standard deviations (SDs), and medians with ranges were reported for continuous variables; frequencies and proportions were reported for categorical variables. Comparisons between cohorts were conducted using the Wilcoxon rank-sum test for continuous variables and chi-squared test for categorical variables.

#### Efficacy analyses

For OS and DOT, the median time-to-event, corresponding 95% CI, and log-rank test *p* values were reported. Unadjusted survival and on-treatment rates at specific time points were obtained using the Nelson–Aalen estimator [31, 32], and unadjusted OS and DOT estimates up to each of these timepoints were obtained using the Kaplan–Meier method.

Comparative analyses of OS, DOT, and change in serum tryptase levels employed a two-step process to obtain an effect estimate that was doubly robust against confounding [33]. First, stabilized inverse-probability-of-treatment-weights (IPTW) were created using logistic regression models, calculated as the inverse of the propensity score, i.e., probability of being in the respective treatment cohort (i.e., avapritinib or BAT), conditional on pre-specified key covariates. Weights were truncated at the 1st and 99th percentiles to reduce variability. Standardized differences were used to assess balance of covariates before and after IPTW weighting, with a difference >10% indicating meaningful imbalance between the two cohorts [34].

Next, IPTW-weighted multivariable Cox proportional hazards models were used to compare survival and DOT and IPTW-weighted multivariable generalized estimating equation linear models were used to compare change in serum tryptase between the avapritinib and BAT cohorts, with further adjustment for key covariates that remained unbalanced after weighting. A two-sided *p* < 0.05 was considered statistically significant without multiplicity adjustment. Robust variance estimation was used to account for the within-subject correlation of BAT cohort patients who contributed multiple lines of therapy, as well as for the application of weights.

#### Safety analyses

AEs that resulted in treatment modification or discontinuation, hospitalization, or death were summarized using descriptive statistics for the BAT cohort.

#### Subgroup and sensitivity analyses

The primary endpoint of OS was compared in the following subgroups: (1) patients who initiated 1L avapritinib at any dose in EXPLORER and PATHFINDER vs. patients who received 1L BAT; (2) all patients who initiated avapritinib at ≤200 mg in EXPLORER and PATHFINDER vs. BAT regardless of the number of prior lines of therapy; (3) patients who received at least one prior systemic therapy (2L + patients) prior to initiating avapritinib at ≤200 mg in EXPLORER and PATHFINDER vs. 2L + BAT patients; (4) 2L + patients who started avapritinib at 200 mg in EXPLORER and PATHFINDER vs. 2L + patients who received BAT; and (5) 2L + patients who started avapritinib at 200 mg in PATHFINDER only vs. 2L + patients who received BAT, using the PATHFINDER safety population and RAC-RE population, respectively. In sensitivity analyses of OS, the impact of excluding patients with missing performance status was evaluated, and index year of treatment was included as a covariate in the Cox model to assess the impact of trends over time in AdvSM care (it was not included in the IPTW model due to inadequate overlap in index year between the avapritinib and BAT cohorts).

### Software

All data cleaning and analyses were conducted using SAS® Enterprise Guide® (version 7.1) and R (version 3.6.3).

## Results

### Study sample

Data were collected from 161 patients who received BAT for AdvSM. After excluding 20 (12.4%) patients with missing performance status, 141 were included in the BAT cohort for comparison with 176 patients in the avapritinib cohort enrolled in EXPLORER (*n* = 69) and PATHFINDER (*n* = 107). While patients in the avapritinib cohort contributed data on a single line of therapy each, the 141 BAT patients contributed 222 lines of therapy. The median number of lines of therapy per BAT patient was 1.0 (range, 1.0–7.0) (Table 1). Of the 222 lines of therapy, 118 (53.2%) were first, 69 (31.1%) second, and 35 (15.8%) third line or later. Across lines, patients were most frequently treated with TKIs (54.1% of lines), followed by cytoreductive therapy (41.0%) and biologic therapies (11.3%). Among 196 lines of therapy with agent-level information available, midostaurin (50.5%) and cladribine (25.0%) were most often used.

**Table 1:** Summary of best available therapies received by real-world patients, overall and by line of therapy.

	Overall^a^	First line	Second line	Third or later lines
Number of unique patients	*N* = 141	*N* = 118	*N* = 69	*N* = 35
Total number of lines of therapy included	*N* = 222	*N* = 118	*N* = 69	*N* = 35
Total number of lines of therapy contributed by patient				
Mean (SD)	1.6 (0.9)	–	–	–
Median (min, max)	1.0 (1.0, 7.0)	–	–	–
Number of lines of therapy contributed, *n* (%)				
1	86 (61.0%)	–	–	–
2	40 (28.4%)	–	–	–
≥3	15 (10.6%)	–	–	–
Year of line of therapy start date, *n* (%)				
2009–2013	66 (29.7%)	–	–	–
2014–2017	99 (44.6%)	–	–	–
2018–2021	57 (25.7%)	–	–	–
Agents used in each included line of therapy, *n* (%)				
TKI therapy	120 (54.1%)	71 (60.2%)	34 (49.3%)	15 (42.9%)
Cytoreductive therapy	91 (41.0%)	39 (33.1%)	33 (47.8%)	19 (54.3%)
Biologic therapy	25 (11.3%)	14 (11.9%)	8 (11.6%)	3 (8.6%)
Agent-level information available^b^	*N* = 196	*N* = 107	*N* = 59	*N* = 30
TKI				
Midostaurin	99 (50.5%)	58 (54.2%)	29 (49.2%)	12 (40.0%)
Ripretinib	4 (2.0%)	2 (1.9%)	0 (0.0%)	2 (6.7%)
Ibrutinib	3 (1.5%)	3 (2.8%)	0 (0.0%)	0 (0.0%)
Dasatinib	2 (1.0%)	1 (0.9%)	1 (1.7%)	0 (0.0%)
Imatinib	2 (1.0%)	1 (0.9%)	0 (0.0%)	1 (3.3%)
Cytoreductive therapy				
Cladribine	49 (25.0%)	20 (18.7%)	18 (30.5%)	11 (36.7%)
Hydroxyurea	17 (8.7%)	10 (9.3%)	5 (8.5%)	2 (6.7%)
Azacitidine	3 (1.5%)	0 (0.0%)	2 (3.4%)	1 (3.3%)
Biologic				
Interferon-alfa	11 (5.6%)	9 (8.4%)	2 (3.4%)	0 (0.0%)
Pegylated interferon	8 (4.1%)	3 (2.8%)	4 (6.8%)	1 (3.3%)
Brentuximab vedotin	4 (2.0%)	2 (1.9%)	2 (3.4%)	0 (0.0%)
Gemtuzumab ozogamicin	1 (0.5%)	0 (0.0%)	0 (0.0%)	1 (3.3%)

*BAT* best available therapy, *ECOG* Eastern Cooperative Oncology Group, *max* maximum, *min* minimum, *SD* standard deviation, *TKI* tyrosine kinase inhibitor.

^a^The BAT cohort was restricted to patients with available ECOG score during any time before to 3 months after the index date.

^b^Agent-level information for prior treatments was reported among patients from all study sites except Medical University of Vienna (Austria) (*N* = 26 lines of therapy), where only treatment class information was collected per local regulations.

#### Baseline characteristics and IPTW weighting

Prior to weighting, region, presence of thrombocytopenia, AdvSM subtype, serum tryptase level ≥125 ng/ml, presence and number of mutated genes within *SRSF2*/*ASXL1*/*RUNX1* gene panel, number of prior lines of therapy, and having received prior TKI therapy were unbalanced between the avapritinib and BAT cohorts (Table 2). The truncated stabilized IPTW weights calculated based on the key baseline covariates had a mean of 0.96 (SD: 0.71; range: 0.46–4.45), indicating the IPTW model was appropriate and stable (Supplementary Table 2). After weighting by truncated stabilized IPTW weights, standardized differences decreased to <10% for most covariates, indicating the two cohorts were more comparable with regards to key covariates (Supplementary Table 3).

**Table 2:** Summary of baseline characteristics.

Baseline characteristics^a^	Avapritinib^b^	BAT^b^	*p*^c^
Number of unique patients	*N* = 176	*N* = 141	
Number of lines of therapy	*N* = 176	*N* = 222	
Demographic characteristics			
Age (years)^d^			0.817
Mean (SD)	66.3 (10.7)	65.5 (11.8)	
Median (min, max)	68.0 (31.0, 88.0)	67.8 (20.9, 87.5)	
Sex, *n* (%)			
Female	73 (41.5%)	76 (34.2%)	0.168
Male	103 (58.5%)	146 (65.8%)	0.168
Region, *n* (%)			
North America	102 (58.0%)	34 (15.3%)	<0.001*
Europe	74 (42.0%)	188 (84.7%)	<0.001*
Medical history			
ECOG Performance status^e^			0.093
*n* (%)	176 (100.0%)	222 (100.0%)	
Mean (SD)	1.2 (0.8)	1.0 (0.7)	
Median (min, max)	1.0 (0.0, 3.0)	1.0 (0.0, 3.0)	
ECOG category, *n* (%)			
0	36 (20.5%)	50 (22.5%)	0.707
1	92 (52.3%)	129 (58.1%)	0.288
≥2	48 (27.3%)	43 (19.4%)	0.081
Anemia^f^, *n* (%)	104 (59.1%)	125 (56.3%)	0.648
Thrombocytopenia^g^, *n* (%)	67 (38.1%)	120 (54.1%)	<0.01*
Disease characteristics			
AdvSM subtype diagnosis,^h^ *n* (%)			
SM-AHN	119 (67.6%)	121 (54.5%)	<0.05*
ASM	29 (16.5%)	68 (30.6%)	<0.01*
MCL	28 (15.9%)	33 (14.9%)	0.883
Any skin involvement, *n* (%)	58 (33.0%)	71 (32.0%)	0.922
Leukocyte count ≥16 × 10^9^/l, *n* (%)	33 (18.8%)	54 (24.3%)	0.225
Serum tryptase level ≥125 ng/ml^i^, *n* (%)	132 (75.0%)	144 (64.9%)	<0.05*
* KIT* mutation^j^			
Patients tested, *n* (%)	170 (96.6%)	140 (99.3%)	0.137
Tested positive for *KIT* D816V, *n* (%)	156 (91.8%)	128 (91.4%)	1.000
* SRSF2*/*ASXL1*/*RUNX1* gene panel^j^			
Patients tested for at least one mutation, *n* (%)	176 (100.0%)	107 (75.9%)	<0.001*
Number of mutated genes within the *SRSF2/ASXL1/RUNX1* gene panel, *n* (%)			
0	92 (52.3%)	41 (38.3%)	0.031
1	54 (30.7%)	44 (41.1%)	0.097
≥2	30 (17.0%)	22 (20.6%)	0.560
Prior therapy			
Patients with prior systemic therapy, *n* (%)	110 (62.5%)	104 (46.8%)	<0.01*
Number of prior systemic therapy lines received, *n* (%)			<0.001*
Mean (SD)	1.0 (1.1)	0.1 (0.3)	
Median (min, max)	1.0 (0.0, 6.0)	0.0 (0.0, 2.0)	
0	66 (37.5%)	118 (53.2%)	<0.01*
1	68 (38.6%)	69 (31.1%)	0.142
2	28 (15.9%)	24 (10.8%)	0.177
≥3	14 (8.0%)	11 (5.0%)	0.309
Prior treatments received, *n* (%)			
TKI therapy	92 (52.3%)	50 (22.5%)	<0.001*
Cytoreductive therapy	33 (18.8%)	61 (27.5%)	0.055
Biologic or other systemic therapy^k^	23 (13.1%)	30 (13.5%)	1.000

*ASM* aggressive systemic mastocytosis, *BAT* best available therapy, *ECOG* Eastern Cooperative Oncology Group, *max* maximum, *min* minimum, *MCL* mast cell leukemia, *SD* standard deviation, *SM-AHN* systemic mastocytosis with associated hematologic neoplasm, *TKI* tyrosine kinase inhibitor.

*p < 0.05.

^a^The baseline period was defined as 8 weeks leading up to the index date for the avapritinib cohort and the 12 weeks leading up to the index date for the BAT cohort.

^b^The trial and real-world samples were restricted to patients with available ECOG score during any time before to 3 months after the index date.

^c^For categorical variables with expected counts <5, Fisher’s exact tests were used instead of chi-squared.

^d^Only the year of birth was collected for the BAT cohort. Patients’ age was calculated using the mid-point of the birth year as approximate dates of birth.

^e^For the BAT cohort, ECOG and Karnofsky scores assessed during 12 months before to 3 months after the index date were considered. For the lines of therapy for which patients had no ECOG score on record during this period (*N* = 9 lines of therapy), the Karnofsky score closest to the index date in the same period was converted to an ECOG score. The conversion was performed according to Oken et al. [37].

^f^For both the avapritinib cohort and the BAT cohort, anemia included reported anemia and hemoglobin <10 g/dl.

^g^For both the avapritinib cohort and the BAT cohort, thrombocytopenia included reported thrombocytopenia and platelet count less than 100 × 10^9^/l.

^h^The AdvSM subtype was assessed at the last diagnosis evaluation prior to or on the index date.

^i^Observations with missing serum tryptase were imputed as not having serum tryptase greater than or equal to 125 ng/ml.

^j^Statistics on *KIT* mutation and *SRSF2/ASXL1/RUNX1* gene panel were reported at the patient level.

^k^Other systemic therapy included steroids and thalidomide or derivatives.

### Main analysis

#### Overall survival

**Fig. 1:**
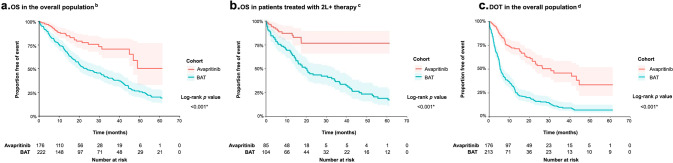
Comparison of OS of patients treated with avapritinib or best available therapy for advanced systemic mastocytosis^a^. The three panels refer to OS among the overall population (**a**), OS among patients treated with 2L+ therapy (**b**), and DOT among the overall population (**c**). 2L+ second or later line of therapy, AdvSM advanced systemic mastocytosis, BAT best available therapy, DOT duration of therapy, OS overall survival. ^a^All Kaplan–Meier curves were truncated at the maximum follow-up of the avapritinib cohort. ^b^A total of 222 lines of therapy were contributed by 141 patients in the BAT cohort. ^c^In the subgroup analysis comparing avapritinib patients treated at ≤200 mg to BAT patients in 2L+, a total of 104 lines of therapy were contributed by 73 patients in BAT cohort. ^d^A total of 213 lines of therapy were contributed by 137 patients in the BAT cohort. Lines of therapy with unknown discontinuation date and unknown last known prescription date were excluded from the analysis of duration of treatment.

In the unweighted sample, there were 34 (19.3%) deaths among 176 avapritinib patients and 84 (59.6%) deaths among 141 BAT patients (Table 3), with a mean follow-up of 17.9 and 25.7 months, respectively. Median OS was not reached (95% CI: 46.9, not estimable) for the avapritinib cohort and was 23.4 (19.5, 32.6) months for the BAT cohort (log-rank *p* < 0.001; Fig. 1a). In adjusted analysis, after weighting, and further adjustment for variables with standardized difference >10% after weighting, OS remained significantly longer in the avapritinib cohort compared with the BAT cohort (IPTW-weighted median OS (95% CI) avapritinib vs. BAT: 49.0 (46.9, not estimable) vs. 26.8 (18.2, 39.7) months; HR (95% CI): 0.48 (0.29, 0.79); *p* = 0.004). The IPTW-weighted OS rates were higher for the avapritinib cohort relative to the BAT cohort across all time points (e.g., 6 months: 96.4% vs. 84.8%; 12 months: 86.4% vs. 73.8%; 24 months: 74.6% vs. 50.9%; 36 months: 68.0% vs. 42.7%) (Table 3). Survival was significantly longer for the avapritinib cohort at all time points tested (*p* < 0.05 for 6 months and subsequent time points), except at 3 months (*p* = 0.087).

**Table 3:** Summary of overall survival.

	Unweighted sample				IPTW-weighted sample^a^			
	Avapritinib	BAT	Estimate (95% CI)	*p*	Avapritinib	BAT	Estimate (95% CI)	*p*
Number of unique patients	*N* = 176	*N* = 141			Effective *N* = 172	Effective *N* = 136		
Number of lines of therapy	*N* = 176	*N* = 222			Effective *N* = 172	Effective *N* = 210		
Deaths of unique patients, *n* (%)	34 (19.3%)	84 (59.6%)	–	–	36 (20.9%)	76 (55.9%)	–	–
Unique patients censored due to avapritinib initiation, *n* (%)	–	21 (14.9%)	–	–	–	25 (18.4%)	–	–
Unique patients censored due to new primary malignancy after the index date, *n* (%)	–	6 (4.3%)	–	–	–	8 (5.9%)	–	–
Mean follow-up (months)	17.9	25.7	–	–	17.9	25.7	–	–
Median overall survival (months) (95% CI)	NR (46.9, NE)	23.4 (19.5, 32.6)	–	–	49.0 (46.9, NE)	26.8 (18.2, 39.7)	–	–
HR (95% CI)^b^	–	–	0.39 (0.26, 0.58)	<0.001*	–	–	0.48 (0.29, 0.79)	0.004*
Survival rate				**Log-rank** ***p***				**Log-rank** ***p***
3 months	97.1%	91.4%	–	0.017*	98.0%	92.8%	–	0.087
6 months	94.7%	83.0%	–	<0.001*	96.4%	84.8%	–	0.006*
9 months	89.7%	77.7%	–	0.001*	89.6%	78.2%	–	0.013*
12 months	87.3%	72.0%	–	<0.001*	86.4%	73.8%	–	0.013*
18 months	80.4%	58.4%	–	<0.001*	79.5%	58.1%	–	<0.001*
24 months	77.5%	49.2%	–	<0.001*	74.5%	50.9%	–	<0.001*
30 months	73.3%	45.5%	–	<0.001*	69.6%	47.6%	–	<0.001*
36 months	70.7%	40.1%	–	<0.001*	67.9%	42.7%	–	<0.001*
48 months	58.7%	26.6%	–	<0.001*	61.9%	30.0%	–	<0.001*
60 months	50.3%	20.2%	–	<0.001*	36.8%	23.4%	–	0.001*

*AdvSM* advanced systemic mastocytosis, *BAT* best available therapy, *CI* confidence interval, *HR* hazard ratio, *IPTW* inverse probability of treatment weighting, *NE* not estimable, *NR* not reached.

*p < 0.05.

^a^Stabilized weights were generated using the following baseline characteristics: age, sex, region, ECOG score, anemia (hemoglobin <10 g/dl), thrombocytopenia (platelet count <100 × 10^9^/l), AdvSM subtype, skin involvement, leukocyte count of 16 × 10^9^/l or higher, serum tryptase level of 125 ng/ml or higher, number of mutated genes within the *SRSF2/ASXL1/RUNX1* gene panel, number of prior lines of therapy, and prior use of tyrosine kinase inhibitor, cytoreductive, biologic or other systemic therapy.

^b^Both unweighted and IPTW-weighted Cox proportional hazards models with a robust sandwich variance estimator were used to model overall survival. IPTW-weighted Cox proportional hazards model further adjusted for covariates with a standardized difference of greater than 10% after weighting, which included region, presence of thrombocytopenia at baseline, and prior use of tyrosine kinase inhibitor therapy, using a doubly robust approach.

In the sensitivity analysis, OS was not significantly different between the BAT efficacy analysis sample (i.e., baseline performance status available prior to initiation in each line of therapy (*N* = 222, 88.8% of 250 prior lines of therapy)) and the BAT full sample with missing baseline performance status (*N* = 250 lines of therapy) (log-rank *p* = 0.33). Unweighted Kaplan–Meier analysis suggested that OS was significantly improved in the avapritinib cohort compared to the full BAT sample (including patients with missing baseline performance scores) (log-rank *p* < 0.001), consistent with the main analysis. In addition, when the main analysis was performed with an indicator for index year as a covariate in the Cox regression model for OS, the results were not different from the main analysis (HR (95% CI): 0.48 (0.27, 0.85); *p* = 0.01).

#### Duration of treatment

The DOT analyses included 176 patients in the avapritinib cohort and 137 patients in the BAT cohort contributing 213 lines of therapy (Table 4 and Fig. 1c). In the unweighted sample, the median DOT was 30.6 (95% CI: 21.4, not estimable) months in the avapritinib cohort and 5.5 (5.1, 7.0) months in the BAT cohort. In the adjusted analysis, DOT remained significantly longer in the avapritinib cohort (IPTW-weighted median (95% CI) avapritinib vs. BAT: 23.8 (20.3, 40.9) vs. 5.4 (5.0, 7.5) months; HR (95% CI): 0.36 (0.26, 0.51); *p* < 0.001). After weighting, the proportion of patients that stayed on treatment was significantly higher for the avapritinib cohort than the BAT cohort across all time points (e.g., 6 months: 85.6% vs. 45.0%; 12 months: 67.7% vs. 32.5%; 18 months: 61.3% vs. 20.4%; all *p* < 0.001).

**Table 4:** Summary of duration of treatment.

	Unweighted sample^a^				IPTW-weighted sample^b^			
	Avapritinib	BAT	Estimate (95% CI)	*p*	Avapritinib	BAT	Estimate (95% CI)	*p*
Number of unique patients	*N* = 176	*N* = 137			Effective *N* = 173	Effective *N* = 131		
Number of lines of therapy	*N* = 176	*N* = 213			Effective *N* = 173	Effective *N* = 201		
Number of discontinued lines of therapy	67 (38.1%)	189 (88.7%)			73 (42.2%)	179 (89.1%)		
Number of censored lines of therapy	109 (61.9%)	24 (11.3%)			100 (57.8%)	22 (10.9%)		
Median DOT (months) (95% CI)	30.6 (21.4, NE)	5.5 (5.1, 7.0)	–	–	23.8 (20.3, 40.9)	5.4 (5.0, 7.5)	–	–
HR (95% CI)^c^			0.30 (0.23, 0.39)	<0.001*			0.36 (0.26, 0.51)	<0.001*
Proportion still on treatment				**Log-rank** ***p***				**Log-rank** ***p***
3 months	94.9%	71.1%	–	<0.001*	95.0%	71.7%	–	<0.001*
6 months	85.4%	46.8%	–	<0.001*	85.6%	45.0%	–	<0.001*
9 months	75.2%	38.2%	–	<0.001*	71.0%	38.3%	–	<0.001*
12 months	72.1%	32.0%	–	<0.001*	67.7%	32.5%	–	<0.001*
18 months	63.5%	20.8%	–	<0.001*	61.3%	20.3%	–	<0.001*

*AdvSM* advanced systemic mastocytosis, *BAT* best available therapy, *CI* confidence interval, *DOT* duration of treatment, *HR* hazard ratio, *IPTW* inverse probability of treatment weighting, *NE* not estimable.

*p < 0.05.

^a^Lines of therapy with unknown discontinuation date and unknown last known prescription date were excluded from the duration of treatment analysis.

^b^Stabilized weights were generated using the following baseline characteristics: age, sex, region, ECOG score, anemia (hemoglobin <10 g/dl), thrombocytopenia (platelet count <100 × 10^9^/l), AdvSM subtype, skin involvement, leukocyte count of 16 × 10^9^/l or higher, serum tryptase level of 125 ng/ml or higher, number of mutated genes within the *SRSF2/ASXL1/RUNX1* gene panel, number of prior lines of therapy, and prior use of tyrosine kinase inhibitor, cytoreductive, biologic or other systemic therapy.

^c^Both unweighted and IPTW-weighted Cox proportional hazards models with a robust sandwich variance estimator were used to model duration of treatment. IPTW-weighted Cox proportional hazards model further adjusted for covariates with a standardized difference of greater than 10% after weighting, which included region, presence of thrombocytopenia at baseline, and prior use of tyrosine kinase inhibitor therapy, using a doubly robust approach.

#### Change in serum tryptase levels

The analysis of 2-month changes in serum tryptase levels included 154 patients in the avapritinib cohort and 43 patients in the BAT cohort (Table 5). The mean percentage change in serum tryptase level from baseline to 2 months was greater for the avapritinib cohort (−71.5% (SD: 35.9%)) than the BAT cohort (37.9% (SD: 269.3%)) (mean difference: −103.0% (95% CI: −167.1%, −38.9%); *p* = 0.002). The same trend was observed after weighting, with a greater mean percentage change in serum tryptase level in the avapritinib cohort (−71.3% (SD: 35.2%)) compared to the BAT cohort (1.7% (148.8%)), although the corresponding mean difference in the percentage change was not estimable by an adjusted linear model due to model non-convergence caused by the small sample size of the BAT cohort.

**Table 5:** Summary of change in serum tryptase levels.

	Unweighted sample^a^				IPTW-weighted sample^b^			
	Avapritinib	BAT	Estimate (95% CI)	*p*	Avapritinib	BAT	Estimate (95% CI)	*p*
Patients included in analysis of change from baseline to 2 months^c^, *n*	154	43			Effective *N* = 150	Effective *N* = 34		
Number of lines of therapy analyzed	154	50			Effective *N* = 150	Effective *N* = 41		
Absolute change								
Mean (SD)	−226.6 (218.9)	−48.6 (299.6)	−168.48 (−257.92, −79.05)	<0.001*	−234.9 (229.8)	−79.8 (209.3)	–	
Median (range)	−164.9 (−1056.0, 543.0)	−8.5 (−1050.0, 1137.7)			−166.3 (−1056.0, 543.0)	−33.0 (−1050.0, 1137.7)		
Percentage change								
Mean (SD)	−71.5 (35.9)	37.9 (269.3)	−103.00 (−167.11, −38.90)	0.002*	−71.3 (35.2)	1.7 (148.8)	–	
Median (range)	−84.5 (−98.9, 129.3)	−12.1 (−94.2, 1826.2)			−84.6 (−98.9, 129.3)	−24.4 (−94.2, 1826.2)		
Maximum reduction	*N* = 175	*N* = 116			Effective *N* = 173	Effective *N* = 106		
Number of lines of therapy analyzed	*N* = 175	*N* = 161			Effective *N* = 173	Effective *N* = 150		
Absolute reduction								
Mean (SD)	−265.9 (232.5)	−108.4 (264.1)	−181.40 (−215.75, −147.04)	<0.001*	−278.4 (245.8)	−114.7 (245.1)	−211.94 (−266.74, −157.14)	<0.001*
Median (range)	−188.7 (−1284.1, −4.5)	−52.3 (−1050.0, 1137.7)			−194.1 (−1284.1, −4.5)	−54.0 (−1050.0, 1137.7)		
Percentage reduction								
Mean (SD)	−86.6 (18.2)	−9.2 (161.4)	−77.86 (−103.43, −52.29)	<0.001*	−87.1 (17.2)	−18.0 (123.9)	−60.34 (−72.81, −47.86)	<0.001*
Median (range)	−92.7 (−99.5, −7.8)	−36.3 (−99.4, 1826.2)			−92.7 (−99.5, −7.8)	−36.9 (−99.4, 1826.2)		
Time to maximum reduction								
Mean (SD)	9.6 (9.7)	7.0 (12.7)			8.8 (9.2)	8.5 (17.1)		
Median (range)	5.6 (0.5, 49.4)	3.2 (0.1, 115.4)			5.6 (0.5, 49.4)	3.2 (0.1, 115.4)		

*AdvSM* advanced systemic mastocytosis, *BAT* best available therapy, *CI* confidence interval, *DOT* duration of treatment, *HR* hazard ratio, *IPTW* inverse probability of treatment weighting, *LOT* line of therapy, *NE* not estimable, *NR* not reached.

*p < 0.05.

^a^LOTs without a tryptase measurement at baseline or in the specified time window and LOTs with unknown discontinuation and last prescription date were excluded from the serum tryptase analyses.

^b^Stabilized weights were generated using the following baseline characteristics: age, sex, region, ECOG score, anemia (hemoglobin <10 g/dl), thrombocytopenia (platelet count <100 × 10^9^/l), AdvSM subtype, skin involvement, leukocyte count of 16 × 10^9^/l or higher, serum tryptase level of 125 ng/ml or higher, number of mutated genes within the *SRSF2/ASXL1/RUNX1* gene panel, number of prior LOTs, and prior use of tyrosine kinase inhibitor therapy.

^c^For the BAT cohort, the serum tryptase level at 2 months was calculated as the serum tryptase level closest to 60 days (±11 days) from the LOT start date, which corresponds to the 3 day 1 of cycle (C3D1) assessment in the EXPLORER and PATHFINDER trials. For avapritinib patients, serum tryptase level at 2 months was defined as the measurement taken at the C3D1 assessment.

The analysis of maximum reduction in serum tryptase included 175 patients in the avapritinib cohort and 116 patients in the BAT cohort (Table 5). In the unweighted sample, the maximum percentage reduction of serum tryptase level was −86.6% (SD: 18.2%) in the avapritinib cohort and −9.2% (161.4%) in the BAT cohort, corresponding to an unadjusted mean difference of −77.9% (95% CI: −103.4%, −52.3%) (*p* < 0.001). After weighting, the avapritinib cohort had a significantly greater maximum reduction in serum tryptase level, with mean difference of −60.3% (95% CI: −72.8%, −47.9%) (*p* < 0.001). The mean time to maximum reduction after weighting was 8.8 (SD: 9.2) months in the avapritinib cohort and 8.5 (17.1) months in the BAT cohort.

#### Safety (BAT cohort)

A total of 250 lines of therapy, contributed by 161 BAT patients, were included in the safety analysis (Table 6). Overall, at least one AE resulting in treatment modification or discontinuation, hospitalization, or death was reported in 100 (40.0%) lines of therapy. In 1L, the most reported AEs were anemia (8.5%) followed by neutropenia (7.1%), while in 2L they were neutropenia and vomiting (5.4% each).

**Table 6:** Summary of safety for the BAT cohort, overall and by line of therapy.

	Overall	First line	Second line	Third or later lines
Unique patients, *n*	161	141	74	24
Lines of therapy (LOTs), *n*	250	141	74	35
AEs that result in treatment modification or discontinuation, hospitalization, or death				
LOTs with any AE, *n* (%)	100 (40.0%)	58 (41.1%)	26 (35.1%)	16 (45.7%)
Mean number of AEs in LOT (SD)	0.6 (1.0)	0.7 (1.1)	0.5 (0.8)	0.8 (1.1)
Median number of AEs in LOT (min, max)	0.0 (0.0, 7.0)	0.0 (0.0, 7.0)	0.0 (0.0, 4.0)	0.0 (0.0, 4.0)
LOTs with 1 AE, *n* (%)	65 (26.0%)	41 (29.1%)	18 (24.3%)	6 (17.1%)
LOTs with 2 AEs, *n* (%)	20 (8.0%)	7 (5.0%)	5 (6.8%)	8 (22.9%)
LOTs with ≥3 AEs, *n* (%)	15 (6.0%)	10 (7.1%)	3 (4.1%)	2 (5.7%)
LOTs with AEs by type, *n* (%)				
Anemia	18 (7.2%)	12 (8.5%)	3 (4.1%)	3 (8.6%)
Nausea	15 (6.0%)	8 (5.7%)	3 (4.1%)	4 (11.4%)
Neutropenia	15 (6.0%)	10 (7.1%)	4 (5.4%)	1 (2.9%)
Thrombocytopenia	11 (4.4%)	6 (4.3%)	1 (1.4%)	4 (11.4%)
Vomiting	8 (3.2%)	3 (2.1%)	4 (5.4%)	1 (2.9%)
Diarrhea	7 (2.8%)	4 (2.8%)	1 (1.4%)	2 (5.7%)
Infection	4 (1.6%)	2 (1.4%)	1 (1.4%)	1 (2.9%)
Fever	3 (1.2%)	1 (0.7%)	2 (2.7%)	0 (0.0%)
Peripheral edema	2 (0.8%)	2 (1.4%)	0 (0.0%)	0 (0.0%)
Abdominal pain	1 (0.4%)	1 (0.7%)	0 (0.0%)	0 (0.0%)
Cough	1 (0.4%)	1 (0.7%)	0 (0.0%)	0 (0.0%)
Fatigue	1 (0.4%)	1 (0.7%)	0 (0.0%)	0 (0.0%)
Dizziness	1 (0.4%)	0 (0.0%)	1 (1.4%)	0 (0.0%)
Intracranial bleeding	1 (0.4%)	0 (0.0%)	1 (1.4%)	0 (0.0%)
Cognitive effects (confusion or memory impairment)	1 (0.4%)	0 (0.0%)	0 (0.0%)	1 (2.9%)
Decreased appetite	1 (0.4%)	0 (0.0%)	0 (0.0%)	1 (2.9%)
Other^a^	50 (20.0%)	27 (19.1%)	14 (18.9%)	9 (25.7%)
Ascites	4 (1.6%)	3 (2.1%)	1 (1.4%)	0 (0.0%)
Pleural effusion	4 (1.6%)	4 (2.8%)	0 (0.0%)	0 (0.0%)

*AE* adverse event, *LOT* line of therapy, *max* maximum, *min* minimum, *SD* standard deviation.

^a^Other AEs occurring in at least 1% of LOTs.

### Analyses of OS among subgroups

The OS comparisons (weighted and unweighted) by patient subgroup are described in Table 7, Fig. 1b, and Supplementary Figs. 1–5. In the adjusted analyses, OS was longer in the avapritinib cohort than in the BAT cohort for all subgroups examined. Specifically, OS was significantly improved in the avapritinib cohorts when comparing patients who received avapritinib vs. BAT as 1L therapy (0.40 (95% CI: 0.22, 0.74); *p* = 0.003); avapritinib (200 mg) vs. BAT as 2L + therapy (0.37 (0.18, 0.75); *p* = 0.006); and avapritinib (≤200 mg) vs. BAT as 2L + therapy (0.34 (0.17, 0.69); *p* = 0.003). Although non-significant, a similar trend of improved OS was observed in the PATHFINDER-only analyses of avapritinib (200 mg) vs. BAT as 2L + therapy (PATHFINDER RAC-RE population: 0.47 (95% CI: 0.21, 1.09), *p* = 0.080; PATHFINDER safety population: 0.49 (0.20, 1.23), *p* = 0.127).

**Table 7:** Summary of overall survival in patient subgroups.

Study sample	Unweighted sample				IPTW-Weighted sample^a^			
	Avapritinib	BAT	Estimate	*p*	Avapritinib	BAT	Estimate	*p*
			(95% CI)^d^				(95% CI)	
Subgroup 1: Avapritinib vs. BAT, 1L								
﻿Number of lines of therapy (number of unique patients)^b^	66 (66)	118 (118)			62 (62)	115 (115)		
Median OS (months) (95% CI)	46.9 (46.9, NE)	27.0 (20.0, 44.3)	–	–	49.0 (29.6, NE)	27.0 (19.7, 44.3)	–	–
HR (95% CI)^c^	–	–	0.50 (0.28, 0.87)	0.014*	–	–	0.40 (0.22, 0.74)^d^	0.003*
Subgroup 2: Avapritinib (≤200 mg) vs. BAT, 1L+								
Number of lines of therapy (number of unique patients)^b^	136 (136)	222 (141)			133 (133)	212 (135)		
Median OS (months) (95% CI)	NR (49.0, NE)	23.4 (19.5, 32.6)	–	–	49.0 (49.0, NE)	26.8 (19.5, 37.2)	–	–
HR (95% CI)^c^	–	–	0.37 (0.23, 0.60)	<0.001*	–	–	0.43 (0.26, 0.72)^e^	0.001*
Subgroup 3: Avapritinib (≤200 mg) vs. BAT, 2L+								
Number of lines of therapy (number of unique patients)^b^	85 (85)	104 (73)			83 (83)	95 (64)		
Median OS (months) (95% CI)	NR (NE, NE)	20.3 (14.9, 33.9)	–	–	NR (NE, NE)	17.9 (14.8, 36.5)	–	–
HR (95% CI)^c^	–	–	0.32 (0.17, 0.60)	<0.001*	–	–	0.34 (0.17, 0.69)^f^	0.003*
Subgroup 4: Avapritinib (200 mg) vs. BAT, 2L+								
Number of lines of therapy (number of unique patients)^b^	79 (79)	104 (73)			77 (77)	96 (66)		
Median OS (months) (95% CI)	NR (NE, NE)	20.3 (14.9, 33.9)	–	–	NR (NE, NE)	17.2 (14.6, 36.5)	–	–
HR (95% CI)^c^	–	–	0.39 (0.21, 0.74)	0.004*	–	–	0.37 (0.18, 0.75)^g^	0.006*
Subgroup 5: Avapritinib PATHFINDER (200 mg) (RAC-RE population) vs. BAT, 2L+								
Number of lines of therapy (number of unique patients)^b^	47 (47)	104 (73)			41 (41)	99 (67)		
Median OS (months) (95% CI)	NR (NE, NE)	20.3 (14.9, 33.9)	–	–	NR (17.5, NE)	17.2 (14.6, 33.9)	–	–
HR (95% CI)^c^	–	–	0.52 (0.26, 1.03)	0.060	–	–	0.47 (0.21, 1.09)^h^	0.080
Subgroup 6: Avapritinib PATHFINDER (200 mg) (Safety population) vs. BAT, 2L+								
Number of lines of therapy (number of unique patients)^b^	67 (67)	104 (73)			66 (66)	97 (67)		
Median OS (months) (95% CI)	NR (NE, NE)	20.3 (14.9, 33.9)	–	–	NR (17.4, NE)	17.9 (14.8, 36.5)	–	–
HR (95% CI)^c^	–	–	0.40 (0.20, 0.81)	0.010*	–	–	0.49 (0.20, 1.23)^i^	0.127

*1L* first line of therapy, *1L+* first or later line of therapy, *2L+* second or later line of therapy, *AdvSM* advanced systemic mastocytosis, *BAT* best available therapy, *CI* confidence interval, *ECOG* Eastern Cooperative Oncology Group, *HR* hazard ratio, *IPTW* inverse probability of treatment weighting, *NE* not estimable, *NR* not reached, *RAC-RE* response assessment committee-response evaluable.

*p < 0.05.

^a^Stabilized weights were generated using the following baseline characteristics: age, sex, region, ECOG score, anemia (hemoglobin <10 g/dl), thrombocytopenia (platelet count <100 × 10^9^/l), AdvSM subtype, skin involvement, leukocyte count of 16 × 10^9^/l or higher, serum tryptase level of 125 ng/ml or higher, number of mutated genes within the *SRSF2/ASXL1/RUNX1* gene panel.

^b^Effective sample size for the number of lines of therapy and number of unique patients were reported for the weighted population.

^c^Both unweighted and IPTW-weighted Cox proportional hazards models with a robust sandwich variance estimator were used to model overall survival. IPTW-weighted Cox proportional hazards model further adjusted for covariates with a standardized difference of greater than 10% after weighting, using a doubly robust approach. HR and the corresponding 95% CI and *p* value were presented. Two-sided *p* value <0.05 was considered statistically significant without multiplicity adjustment.

^d^IPTW-weighted multivariable Cox proportional hazards model further adjusted for age, ECOG score, AdvSM subtype, and skin involvement.

^e^IPTW-weighted multivariable Cox proportional hazards model further adjusted for region, presence of thrombocytopenia at baseline, serum tryptase level of 125 ng/ml or higher, and prior use of tyrosine kinase inhibitor therapy.

^f^IPTW-weighted multivariable Cox proportional hazards model further adjusted for sex, region, ECOG score, presence of thrombocytopenia at baseline, leukocyte count of 16 × 10^9^/l or higher, prior use of tyrosine kinase inhibitor therapy, and prior use of cytoreductive therapy.

^g^IPTW-weighted multivariable Cox proportional hazards model further adjusted for sex, region, ECOG score, presence of thrombocytopenia at baseline, leukocyte count of 16 × 10^9^/l or higher, serum tryptase level of 125 ng/ml or higher, prior use of tyrosine kinase inhibitor therapy, and prior use of cytoreductive therapy.

^h^IPTW-weighted multivariable Cox proportional hazards model further adjusted for sex, region, presence of anemia at baseline, presence of thrombocytopenia at baseline, AdvSM subtype, prior use of tyrosine kinase inhibitor therapy, and prior use of cytoreductive therapy.

^i^IPTW-weighted multivariable Cox proportional hazards model further adjusted for age, region, ECOG score, presence of thrombocytopenia at baseline, leukocyte count of 16 × 10^9^/l or higher, serum tryptase level of 125 ng/ml or higher, and prior use of tyrosine kinase inhibitor therapy.

## Discussion

This study collected data via a retrospective chart review on patients with AdvSM receiving BAT to serve as controls to patients with AdvSM who received avapritinib in the single-arm EXPLORER and PATHFINDER trials. In the absence of a randomized controlled trial, this study presents a valuable perspective on the comparative efficacy of avapritinib compared to BAT in a real-world setting.

After adjusting for differences in key prognostic factors and confounders between the two treatment cohorts, avapritinib was associated with significantly improved survival compared with BAT (HR (95% CI): 0.48 (0.29, 0.79); *p* = 0.004). In subgroup analyses, all subgroups of patients in the avapritinib cohort experienced a reduced risk of death compared to patients in the BAT cohort, with most comparisons statistically significant. Specifically, patients treated in 2L + with avapritinib at a dose of ≤200 mg had decreased risk of death by 66% compared to BAT. The small sample size of certain subgroups may have contributed to statistically non-significant findings. Avapritinib also offered significantly greater reduction in mast cell burden relative to BAT, with median maximum percentage reduction in serum tryptase levels of 93% compared with 37% for BAT. The results of this study, which collected patient-level data allowing for rigorous statistical analysis, further reinforce recent findings from an indirect treatment comparison of avapritinib vs. midostaurin, which compared aggregate-level data from the clinical trials and reported an adjusted HR for OS of 0.44 (95% CI: 0.25–0.76) [35].

Outcomes observed for the BAT cohort in this study are consistent with prior studies of therapies for AdvSM. In a registry-based analysis, Lübke et al. reported an OS from initiation of 1L treatment with midostaurin of 3.1 years (37 months) and OS from initiation of 1L treatment with cladribine of 1.6 years (18 months) [13]. In this study, the most commonly observed 1L therapies in the BAT cohort were midostaurin (54.2%) and cladribine (18.7%), and the mean OS from initiation of 1L treatment for the BAT cohort was 27 months, consistent with findings of Lübke et al. Jawhar et al. reported a median OS of 30 months in a study of midostaurin in 35 patients with AdvSM [16], and a larger open-label study of 116 patients by Gotlib et al. reported a median OS of 34 months [9]. The median best percentage change in serum tryptase levels in the IPTW-weighted BAT cohort was −36.9%, which is generally consistent with prior studies of midostaurin (−58% in Gotlib et al. [9] and −47% in DeAngelo et al. [15]), with differences potentially attributable to the inclusion of therapies other than midostaurin, such as cladribine, in the BAT cohort.

This study benefited from several strengths related to its methodology and employed strategies to maximize comparability between the two cohorts. These included using eligibility criteria for the BAT cohort similar to those of the EXPLORER and PATHFINDER trials, a standardized procedure for data collection across study sites, and harmonization of definitions for the outcomes and key baseline characteristics between the two treatment cohorts. Additionally, patients in the BAT cohort contributed data on multiple lines of therapy, which allowed the statistical analysis to have increased power. Finally, rigorous statistical methods such as IPTW-weighting and doubly robust estimation were used to account for the potential differences in the comprehensive list of a priori specified key adjustment covariates between the avapritinib and BAT cohorts.

The results of this study should be interpreted within the bounds of certain limitations. First, AdvSM diagnosis information collected for the BAT cohort was based on local clinician-assessed evaluation using the 2016 revision to the World Health Organization diagnostic criteria, and correct diagnosis might not have been made prior to the substantial increases in disease awareness and knowledge occurring in last decade. AdvSM diagnoses for the avapritinib cohort were based on the same criteria but confirmed by the RAC. Thus, there may have been misclassification of the clinician-assessed AdvSM diagnosis in the BAT cohort, which could result in an underestimation of the difference in OS (OS for patients with indolent SM and smoldering SM is typically longer than for patients with AdvSM) [7, 36]. However, as all participating sites are centers with expertise in the treatment of AdvSM, this concern is mitigated.

Second, AEs were described for the BAT cohort only, due to differences in definitions and data collection of AEs in EXPLORER/PATHFINDER and non-protocol clinical practice. AEs in the BAT cohort (collected retrospectively from patient charts) are expected to be underreported compared to per-protocol, prospectively collected AEs in a trial setting. In addition, trial AEs are graded per Common Terminology Criteria for Adverse Events, whereas AEs in standard clinical practice are not. This makes comparison (even non-statistical) infeasible.

Third, due to the retrospective nature of data collection for the BAT cohort, the results may have been impacted by incomplete reporting for key characteristics, such as performance status. However, a sensitivity analysis assessing the impact of missing performance status indicated that this is not expected to impact the results.

Lastly, in the primary analysis of OS, 21 (14.9%) patients from the BAT cohort went on to receive avapritinib as part of EXPLORER or PATHFINDER. These patients were included in the BAT cohort and censored at the initiation of avapritinib. Because no identifiable information was collected for real-world patients, some of these patients may have been included in the avapritinib cohort as well. These patients were censored at avapritinib initiation to ensure their time was not counted in both treatment cohorts. Additionally, given the low proportion of cross-over and resulting low impact of bias due to potential informative censoring, additional methods such as inverse probability of censoring weighting were not used for adjustment.

In conclusion, the results from this analysis show that patients with AdvSM treated with avapritinib in EXPLORER and PATHFINDER experienced significantly improved survival, longer DOT, as well as greater reductions in serum tryptase levels, compared to patients treated with BAT. Furthermore, the findings indicated a survival benefit in patients treated with avapritinib at doses of ≤200 mg across all lines compared to BAT. These data offer important insights into the superior comparative efficacy of avapritinib relative to other therapies for AdvSM.

## Supplementary information

Supplemental Material
