# Supplementary material for: Efficacy of avapritinib versus best available therapy in the treatment of advanced systemic mastocytosis
Source: Leukemia. 2022 Jul 5;36(8):2108–20. doi: 10.1038/s41375-022-01615-z (PMC9343245; doi:10.1038/s41375-022-01615-z)
Supplement: Supplementary file 1 — Supplemental Material [file 41375_2022_1615_MOESM1_ESM.docx]

# Supplemental Material

**Supplemental Table 1. Inclusion and exclusion criteria for the external control cohort**

| **External control cohort** |
| --- |
| ***Inclusion Criteria*** |
| Age ≥ 18 years |
| Diagnosed with AdvSM, with known subtype including:   - ASM - SM-AHN - MCL |
| Received at least one line of systemic therapy for AdvSM, which may include but not limited to regimens containing:   - Multikinase inhibitor: midostaurin - Cytoreductive therapy: cladribine, interferon alfa, azacitidine, decitabine - Hydroxyurea - Selective Tyrosine kinase inhibitors (TKIs): imatinib, nilotinib, dasatinib - ADC: brentuximab vedotin, gemtuzumab ozogamicin |
| ***Exclusion Criteria*** |
| History of another primary malignancy that has been diagnosed or required therapy within 3 years prior to the index date (initiation of first systemic line of therapy for AdvSM at the participating site). The following are exempt from the 3-year limit: completely resected basal cell and squamous cell skin cancer, curatively treated localized prostate cancer, and completely resected carcinoma in situ of any site. |
| Among patients with SM-AHN, those in whom any of the following is true:   - the SM component is consistent with indolent systemic mastocytosis (ISM) or smoldering SM (SSM), or - the AHN component is a lymphoid malignancy, or one of the following myeloid malignancies: acute myeloid leukemia (AML), myelodysplastic syndrome (MDS) that is very high- or high-risk as defined by the IPSS-R, or a Philadelphia chromosome positive malignancy, or - there is a known *FIP1L1/PDGFRA* fusion gene (including those with CHIC-2 deletion and partial deletion of *PDGFRA*), independent of *KIT* mutational status.   Patients with known *FIP1L1/PDGFRA* fusion gene (including those with CHIC-2 deletion and partial deletion of *PDGFRA*), independent of *KIT* mutational status, are excluded because they typically have SM associated with a hypereosinophilic syndrome or chronic eosinophilic leukemia, and show a 100% rate of complete response to imatinib, and thus their inclusion may bias the results of this study. |
| Received avapritinib as the first line of systemic therapy for AdvSM at participating site, or prior to initiation of first systemic therapy at participating site. |

**Abbreviations:** ADC: antibody-drug conjugate; AdvSM: advanced systemic mastocytosis; AHN: associated hematologic neoplasm; AML: acute myeloid leukemia; ASM: aggressive systemic mastocytosis; IPSS-R: Revised International Prognostic Scoring System; ISM: indolent systemic mastocytosis; MCL: mast cell leukemia; MDS: myelodysplastic syndrome; SM: systemic mastocytosis; SSM: smoldering systemic mastocytosis; SM-AHN: systemic mastocytosis with associated hematologic neoplasm; TKI: tyrosine kinase inhibitor.

**Supplemental Table 2. Summary Statistics of Truncated Stabilized Weights for Inverse Probability of Treatment Weighting Analysis of Overall Survival**

| **Study sample** | **N** | **Mean (SD)** | | **Min** | **Max** |
| --- | --- | --- | --- | --- | --- |
| Overall^a^ | 389 | 0.98 | (0.84) | 0.47 | 5.81 |
| Avapritinib cohort | 176 | 0.96 | (0.80) | 0.47 | 5.81 |
| Best available therapy cohort | 213 | 0.99 | (0.87) | 0.56 | 5.81 |

**Abbreviations:** Max: maximum; min: minimum; SD: standard deviation.

**Note:**

^a^ Stabilized weights were truncated at the 1st and 99th percentiles.

**Supplemental Table 3. Summary of Baseline Characteristics Before and After Inverse Probability of Treatment Weighting**

| **Baseline characteristics^a^** | | | **Unweighted sample** | | | **IPTW-Weighted sample^b^** | | |
| --- | --- | --- | --- | --- | --- | --- | --- | --- |
|  |  |  | **Avapritinib^c^** | **BAT^c^** | **Standardized Difference^d^** | **Avapritinib^c^** | **BAT^c^** | **Standardized Difference^d^** |
| **Number of unique patients** | | | **N = 176** | **N = 141** |  | **Effective**  **N = 172** | **Effective**  **N = 134** |  |
| **Number of lines of therapy** | | | **N = 176** | **N = 222** |  | **Effective**  **N = 172** | **Effective**  **N = 210** |  |
| **Demographic characteristics** | | |  |  |  |  |  |  |
| **Age (years)^e^** | | |  |  | 6.5% |  |  | 9.2% |
| Mean (SD) | | | 66.3 (10.7) | 65.5 (11.8) |  | 66.4 (10.5) | 65.3 (12.4) |  |
| Median (min, max) | | | 68.0 (31.0, 88.0) | 67.8 (20.9, 87.5) |  | 68.0 (31.0, 88.0) | 67.9 (20.9, 87.5) |  |
| **Sex, n (%)** | | |  |  | 15.0%* |  |  | 5.3% |
| Female | | | 73 (41.5%) | 76 (34.2%) |  | 40.0% | 37.4% |  |
| Male | | | 103 (58.5%) | 146 (65.8%) |  | 60.0% | 62.6% |  |
| **Region, n (%)** | | |  |  | 98.7%* |  |  | 12.3%* |
| North America | | | 102 (58.0%) | 34 (15.3%) |  | 34.4% | 28.6% |  |
| Europe | | | 74 (42.0%) | 188 (84.7%) |  | 65.6% | 71.4% |  |
| **Medical history** | | |  |  |  |  |  |  |
| **Performance status** | | |  |  |  |  |  |  |
| **ECOG^f^** | | |  |  |  |  |  |  |
| n (%) | | | 176 (100.0%) | 222 (100.0%) |  | 100.0% | 100.0% |  |
| Mean (SD) | | | 1.2 (0.8) | 1.0 (0.7) |  | 1.2 (0.8) | 1.1 (0.7) |  |
| Median (min, max) | | | 1.0 (0.0, 3.0) | 1.0 (0.0, 3.0) |  | 1.0 (0.0, 3.0) | 1.0 (0.0, 3.0) |  |
| ECOG category, n (%) | | |  |  |  |  |  |  |
| 0 | | | 36 (20.5%) | 50 (22.5%) | 5.0% | 16.3% | 19.2% | 7.4% |
| 1 | | | 92 (52.3%) | 129 (58.1%) | 11.8%* | 59.0% | 56.2% | 5.8% |
| ≥2 | | | 48 (27.3%) | 43 (19.4%) | 18.8%* | 24.6% | 24.7% | 0.1% |
| **Anemia,^g^ n (%)** | | | 104 (59.1%) | 125 (56.3%) | 5.6% | 55.4% | 57.8% | 5.0% |
| **Thrombocytopenia,^h^ n (%)** | | | 67 (38.1%) | 120 (54.1%) | 32.5%* | 38.9% | 43.9% | 10.2%* |
| **Disease characteristics** | | |  |  |  |  |  |  |
| **AdvSM subtype diagnosis,^i^ n (%)** | | |  |  |  |  |  |  |
| SM-AHN | | | 119 (67.6%) | 121 (54.5%) | 27.1%* | 58.4% | 58.2% | 0.5% |
| ASM | | | 29 (16.5%) | 68 (30.6%) | 33.8%* | 26.5% | 25.2% | 3.0% |
| MCL | | | 28 (15.9%) | 33 (14.9%) | 2.9% | 15.1% | 16.6% | 4.3% |
| **Skin involvement** | | |  |  |  |  |  |  |
| Any skin involvement, n (%) | | | 58 (33.0%) | 71 (32.0%) | 2.1% | 30.3% | 32.5% | 4.8% |
| **Leukocyte count** | | |  |  |  |  |  |  |
| ≥16 × 10^9^/L, n (%) | | | 33 (18.8%) | 54 (24.3%) | 13.6%* | 18.5% | 19.8% | 3.3% |
| **Serum tryptase^j^ (ng/mL)** | | |  |  |  |  |  |  |
| ≥125 ng/mL, n (%) | | | 132 (75.0%) | 144 (64.9%) | 22.2%* | 72.5% | 71.0% | 3.2% |
| ***SRSF2/ASXL1/RUNX1* (S/A/R) mutation panel** | | |  |  |  |  |  |  |
| Number that were tested for at least one mutation, n (%) | | | 176 (100.0%) | 169 (76.1%) |  | 100.0% | 70.8% |  |
| Number of mutated genes within S/A/R panel, n (%) | | |  |  |  |  |  |  |
| 0 | | | 92 (52.3%) | 66 (29.7%) |  | 55.3% | 26.7% |  |
| 1 | | | 54 (30.7%) | 68 (30.6%) | 0.1% | 28.7% | 30.1% | 3.1% |
| ≥2 | | | 30 (17.0%) | 35 (15.8%) | 3.5% | 16.0% | 13.9% | 5.8% |
| **Prior therapy** | | |  |  |  |  |  |  |
| **Prior systemic therapy** | | |  |  |  |  |  |  |
| Patients with prior systemic therapy, n (%) | | | 110 (62.5%) | 104 (46.8%) |  | 52.8% | 49.6% |  |
| **Number of prior lines of systemic therapy received, n (%)** | | |  |  |  |  |  |  |
| Mean (SD) | | | 1.0 (1.1) | 0.1 (0.3) |  | 0.8 (1.0) | 0.1 (0.3) |  |
| Median (min, max) | | | 1.0 (0.0, 6.0) | 0.0 (0.0, 2.0) |  | 1.0 (0.0, 6.0) | 0.0 (0.0, 2.0) |  |
| 0 | | | 66 (37.5%) | 118 (53.2%) | 31.8%* | 47.2% | 50.4% | 6.4% |
| 1 | | | 68 (38.6%) | 69 (31.1%) | 15.9%* | 33.1% | 32.4% | 1.5% |
| 2 | | | 28 (15.9%) | 24 (10.8%) | 15.0%* | 14.6% | 12.6% | 5.6% |
| ≥3 | | | 14 (8.0%) | 11 (5.0%) | 12.2%* | 5.1% | 4.6% | 2.7% |
| **Prior treatments received, n (%)** | | |  |  |  |  |  |  |
| TKI therapy | | | 92 (52.3%) | 50 (22.5%) | 64.6%* | 37.1% | 29.9% | 15.2%* |
| Cytoreductive therapy | | | 33 (18.8%) | 61 (27.5%) | 20.8%* | 20.1% | 22.1% | 4.8% |
| Biologic or other systemic therapy^k^ | | | 23 (13.1%) | 30 (13.5%) | 1.3% | 14.9% | 15.2% | 0.7% |
| **Agent-level information available^l^** | | | **N = 176** | **N = 196** |  | **Effective**  **N = 172** | **Effective**  **N = 193** |  |
| TKI | | |  |  |  |  |  |  |
| Midostaurin | | | 81 (46.0%) | 32 (16.3%) |  | 33.7% | 21.9% |  |
| Dasatinib | | | 6 (3.4%) | 7 (3.6%) |  | 1.9% | 3.6% |  |
| Ibrutinib | | | 2 (1.1%) | 0 (0.0%) |  | 0.5% | 0.0% |  |
| Imatinib | | | 10 (5.7%) | 10 (5.1%) |  | 3.2% | 7.2% |  |
| Nilotinib | | | 2 (1.1%) | 0 (0.0%) |  | 0.8% | 0.0% |  |
| Ripretinib | | | 5 (2.8%) | 1 (0.5%) |  | 1.6% | 0.4% |  |
| Ruxolitinib | | | 2 (1.1%) | 0 (0.0%) |  | 0.6% | 0.0% |  |
| Cytoreductive therapy | | |  |  |  |  |  |  |
| Cladribine | | | 22 (12.5%) | 34 (17.3%) |  | 15.6% | 13.6% |  |
| Azacitidine | | | 5 (2.8%) | 2 (1.0%) |  | 1.9% | 0.9% |  |
| Decitabine | | | 2 (1.1%) | 2 (1.0%) |  | 0.7% | 1.7% |  |
| Chlorambucil | | | 1 (0.6%) | 0 (0.0%) |  | 0.3% | 0.0% |  |
| Hydroxyurea | | | 9 (5.1%) | 17 (8.7%) |  | 3.7% | 7.0% |  |
| Biologic | | |  |  |  |  |  |  |
| Brentuximab vedotin | | | 3 (1.7%) | 2 (1.0%) |  | 1.2% | 3.1% |  |
| Obinituzumab | | | 1 (0.6%) | 0 (0.0%) |  | 0.3% | 0.0% |  |
| Rituximab | | | 1 (0.6%) | 0 (0.0%) |  | 0.3% | 0.0% |  |
| Interferon-alfa | | | 14 (8.0%) | 20 (10.2%) |  | 11.1% | 9.1% |  |
| Pegylated interferon | | | 3 (1.7%) | 8 (4.1%) |  | 2.7% | 4.3% |  |

*Standardized difference greater than 10%.

**Abbreviations:** AdvSM: advanced systemic mastocytosis; ASM: aggressive systemic mastocytosis; BAT: best available therapy; ECOG: Eastern Cooperative Oncology Group; IPTW: inverse probability of treatment weighting; max: maximum; MCL: mast cell leukemia; min: minimum; S/A/R: *SRSF2/ASXL1/RUNX1*; SD: standard deviation; SM-AHN: systemic mastocytosis with an associated hematologic neoplasm; TKI: tyrosine kinase inhibitor.

**Notes:**

^a^ The baseline period was defined as 8 weeks leading up to the index date for the avapritinib cohort and the 12 weeks leading up to the index date for the BAT cohort.

^b^ Stabilized IPTW weights accounted for age, sex, region, ECOG score, anemia (hemoglobin less than 10 g/dL), thrombocytopenia (platelet count less than 100 x 10^9^/L), AdvSM subtype, skin involvement, leukocyte count of 16 × 10^9^ per L or higher, serum tryptase level of 125 ng/mL or higher, number of mutated genes within the *SRSF2/ASXL1/RUNX1* gene panel, number of prior lines of therapy, and types of prior therapy.

^c^ The trial and real-world samples were restricted to patients with available ECOG score during any time before to 3 months after the index date.

^d^ For continuous variables, the standardized difference was calculated by dividing the absolute difference in means of avapritinib cohort vs. BAT cohort by the pooled standard deviation of both cohorts. The pooled standard deviation was the square root of the average of the squared standard deviations. For categorical variables with 2 levels, the standardized difference was calculated using the following equation where P_1_ was the respective proportion of avapritinib cohort, and P_2_ was the respective proportion of BAT cohort: |P_1_-P_2_|/√p(1-p)], where p = (P_1_+P_2_)/2. For each variable, a standardized difference greater than 10% was indicative of meaningful imbalance between the two cohorts, per Austin and Stuart (2015),^33^ and were denoted with "*".

^e^ Only the year of birth was collected for the BAT cohort. Patients' age was calculated using the mid-point of the birth year as approximate dates of birth.

^f^ For the BAT cohort, ECOG and Karnofsky scores assessed during 12 months before to 3 months after the index date were considered. For the lines of therapy for which patients had no ECOG score on record during this period (N = 9 lines of therapy), the Karnofsky score closest to the index date in the same period was converted to an ECOG score. The conversion was performed according to Oken et al.^36^

^g^ For both the avapritinib cohort and the BAT cohort, anemia included reported anemia and hemoglobin less than 10 g/dL.

^h^ For both the avapritinib cohort and the BAT cohort, thrombocytopenia included reported thrombocytopenia and platelet count less than 100 x 10^9^/L.

^i^ The AdvSM subtype was assessed at the last diagnosis evaluation prior to or on the index date.

^j^ Observations with missing serum tryptase level were imputed as not having serum tryptase level greater than or equal to 125 ng/mL.

^k^ Other systemic therapy included steroids and thalidomide or derivatives.

^l^ Agent-level information for prior treatments was reported among patients from all study sites except Medical University of Vienna (Austria) (N=26 lines of therapy), where only treatment class information was collected per local regulations.

**Supplemental Figure 1. Unweighted Kaplan-Meier Curve for Overall Survival for AdvSM:^a^ Avapritinib vs. BAT, 1L**


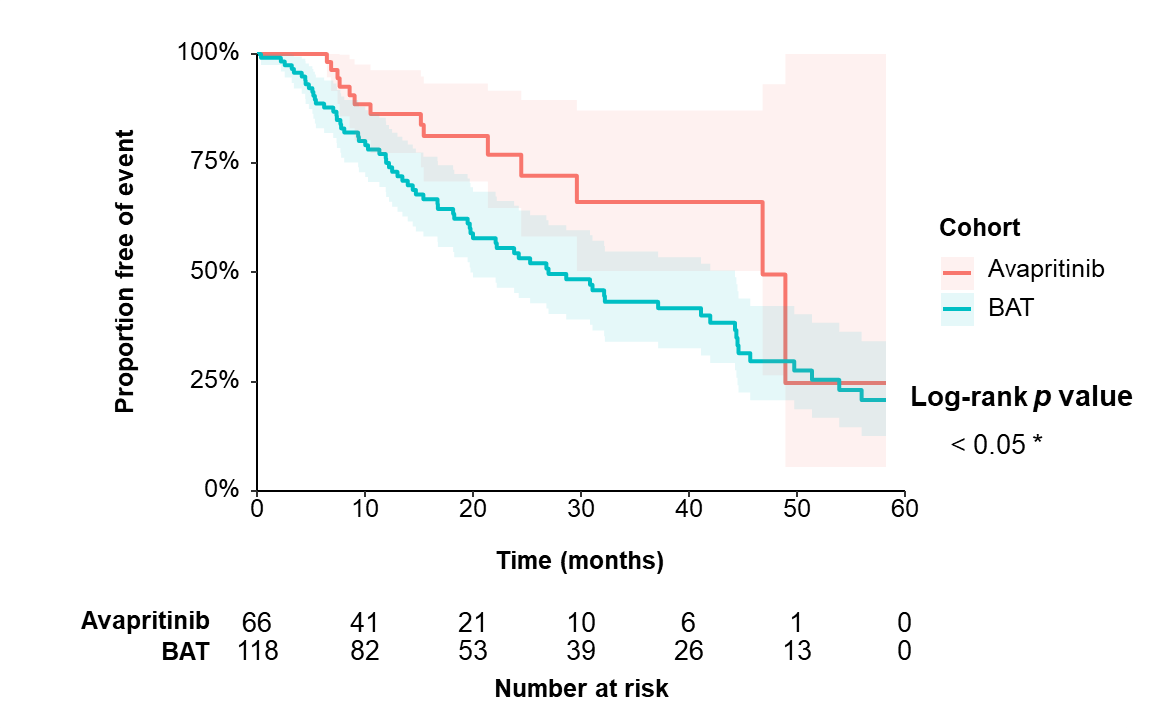


**p*<0.05.

**Abbreviations**: 1L: first line of therapy; AdvSM: advanced systemic mastocytosis; BAT: best available therapy.

**Note:**

^a^ A total of 66 lines of therapy were contributed by 66 trial patients in the unweighted avapritinib cohort. A total of 118 lines of therapy were contributed by 118 real-world patients in the unweighted BAT cohort. The Kaplan-Meier curve was truncated at the maximum follow-up of the avapritinib cohort.

**Supplemental Figure 2. Unweighted Kaplan-Meier Curve for Overall Survival for AdvSM:^a^ Avapritinib (≤200mg) vs. BAT, 1L+**


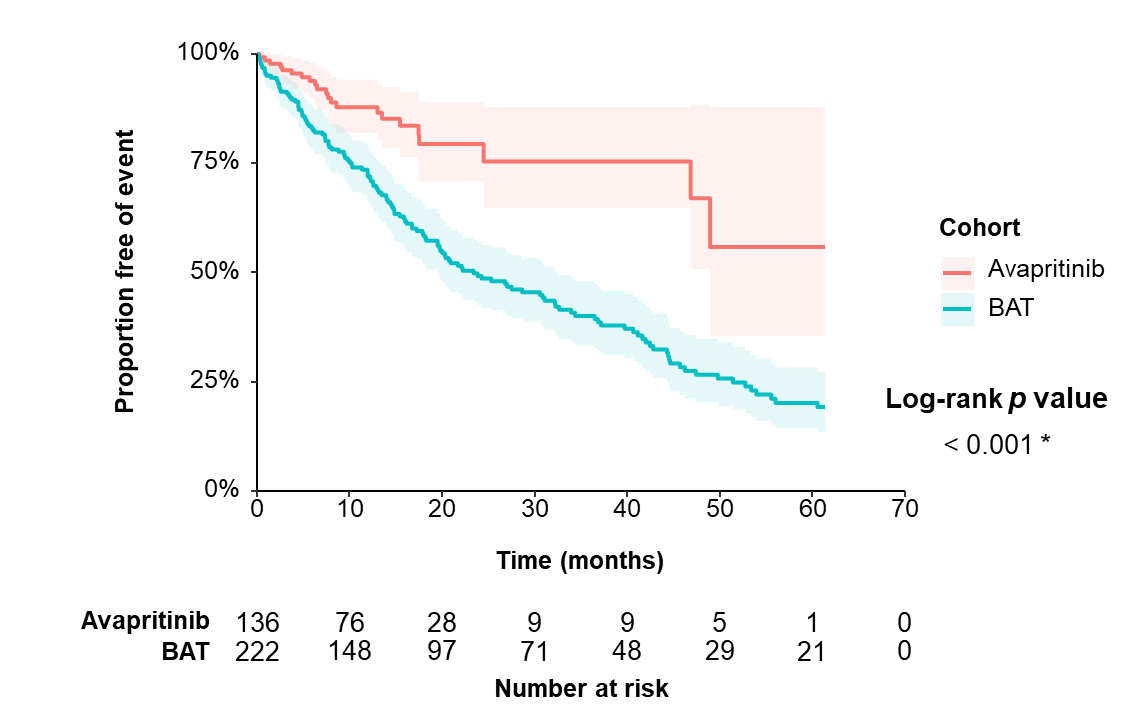


**p*<0.05.

**Abbreviations**: 1L+: first or later line of therapy; AdvSM: advanced systemic mastocytosis; BAT: best available therapy.

**Note:**

^a^ A total of 136 lines of therapy were contributed by 136 trial patients in the unweighted avapritinib cohort. A total of 222 lines of therapy were contributed by 141 real-world patients in the unweighted BAT cohort. The Kaplan-Meier curve was truncated at the maximum follow-up of the avapritinib cohort.

**Supplemental Figure 3. Unweighted Kaplan-Meier Curve for Overall Survival for AdvSM:^a^ Avapritinib (200mg) vs. BAT, 2L+**


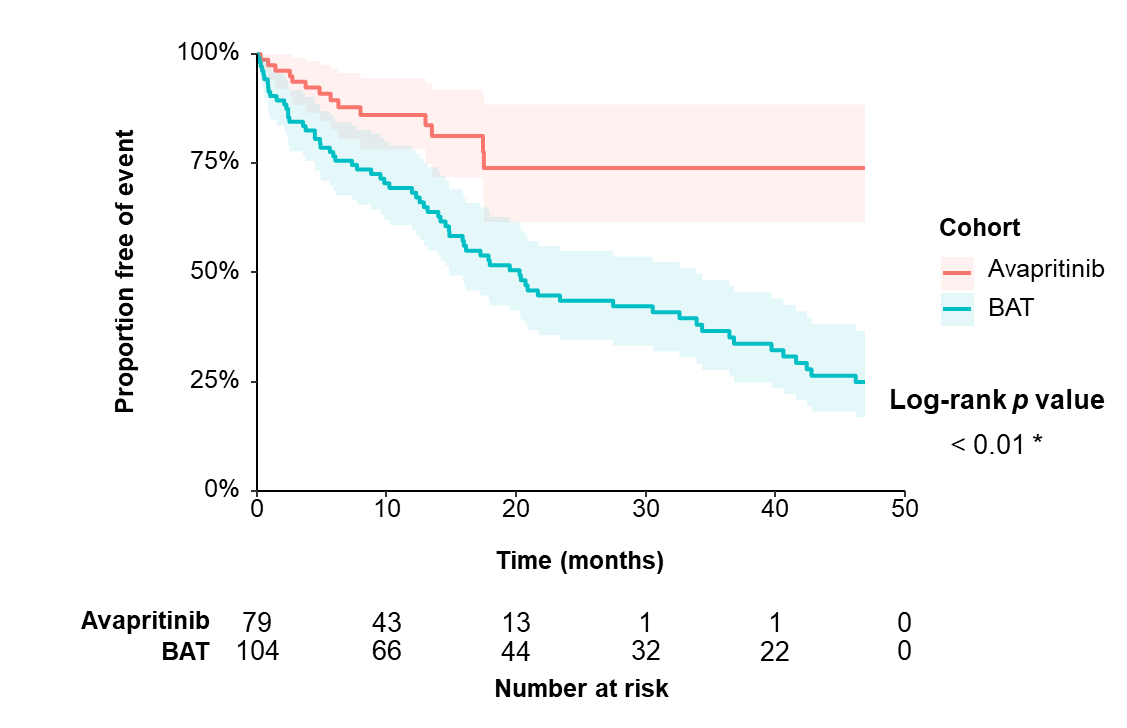


**p*<0.05.

**Abbreviations**: 2L+: second or later line of therapy; AdvSM: advanced systemic mastocytosis; BAT: best available therapy.

**Note:**

^a^ A total of 79 lines of therapy were contributed by 79 trial patients in the unweighted avapritinib cohort. A total of 104 lines of therapy were contributed by 73 real-world patients in the unweighted BAT cohort. The Kaplan-Meier curve was truncated at the maximum follow-up of the avapritinib cohort.

**Supplemental Figure 4. Unweighted Kaplan-Meier Curve for Overall Survival for AdvSM:^a^ Avapritinib PATHFINDER (200mg) (RAC-RE population) vs. BAT, 2L+**


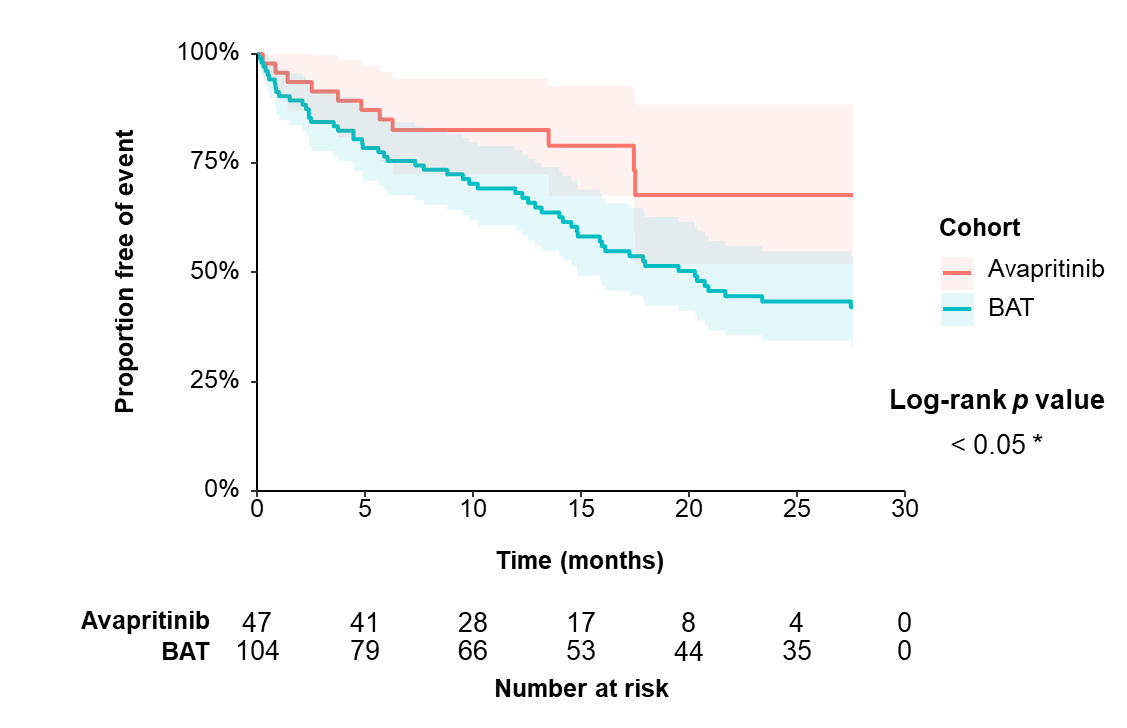


**p*<0.05.

**Abbreviations**: 2L+: second or later line of therapy; AdvSM: advanced systemic mastocytosis; BAT: best available therapy; RAC-RE: response assessment committee adjudicated response-evaluable.

**Note:**

^a^ A total of 47 lines of therapy were contributed by 47 trial patients in the unweighted avapritinib cohort. A total of 104 lines of therapy were contributed by 73 real-world patients in the unweighted BAT cohort. The Kaplan-Meier curve was truncated at the maximum follow-up of the avapritinib cohort.

**Supplemental Figure 5. Unweighted Kaplan-Meier Curve for Overall Survival for AdvSM:^a^ Avapritinib PATHFINDER (200mg) (safety population) vs. BAT, 2L+**


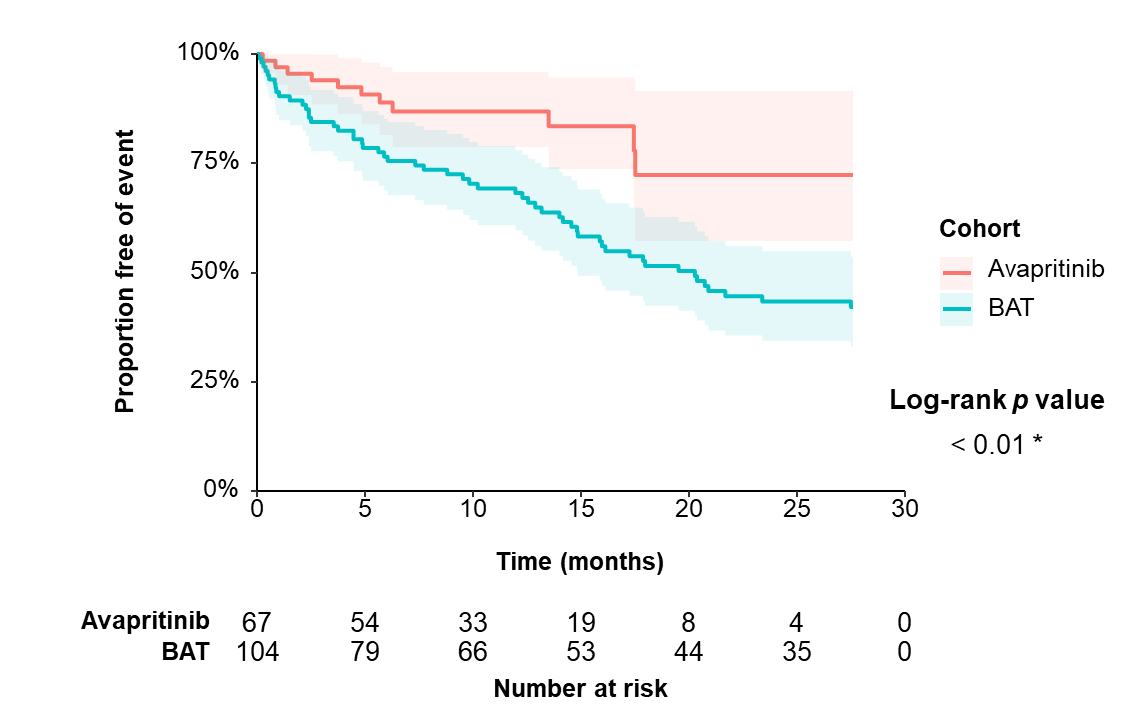


**p*<0.05.

**Abbreviations**: 2L+: second or later line of therapy; AdvSM: advanced systemic mastocytosis; BAT: best available therapy.

**Note:**

^a^ A total of 67 lines of therapy were contributed by 67 trial patients in the unweighted avapritinib cohort. A total of 104 lines of therapy were contributed by 73 real-world patients in the unweighted BAT cohort. The Kaplan-Meier curve was truncated at the maximum follow-up of the avapritinib cohort.
